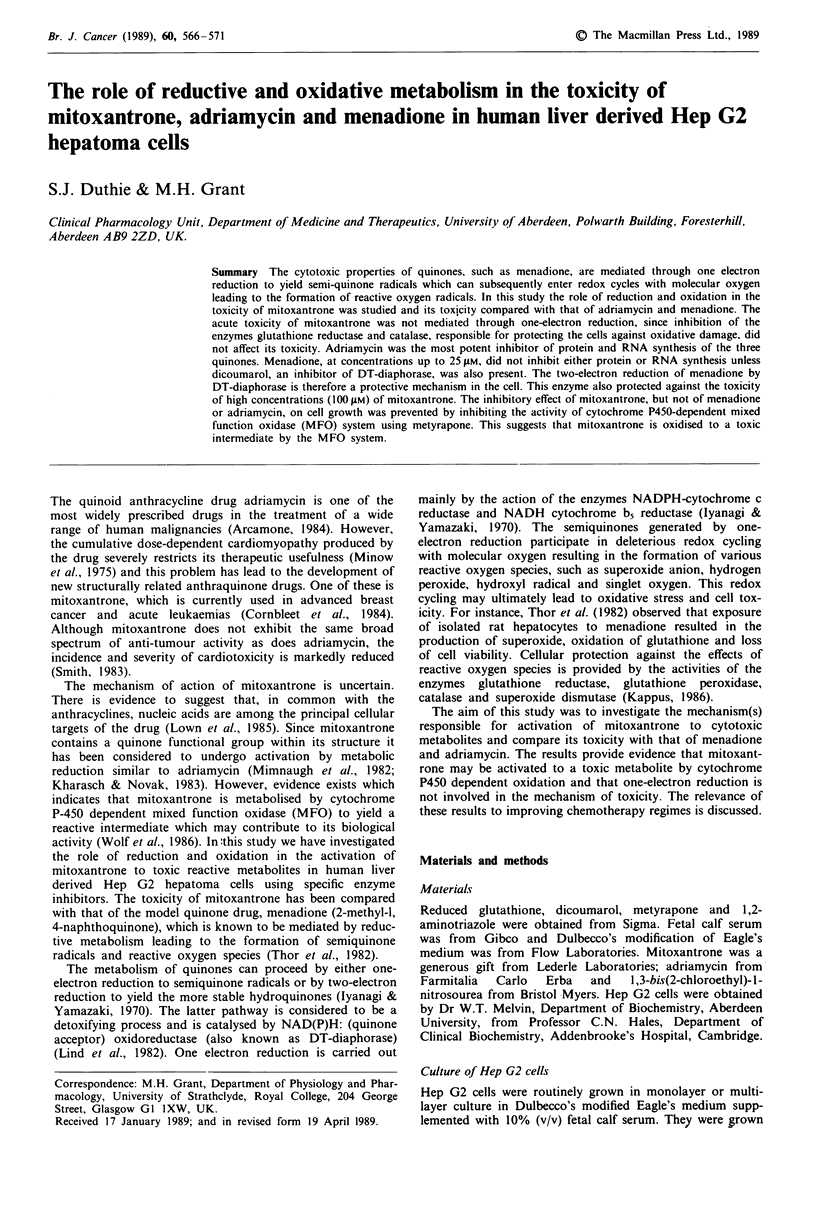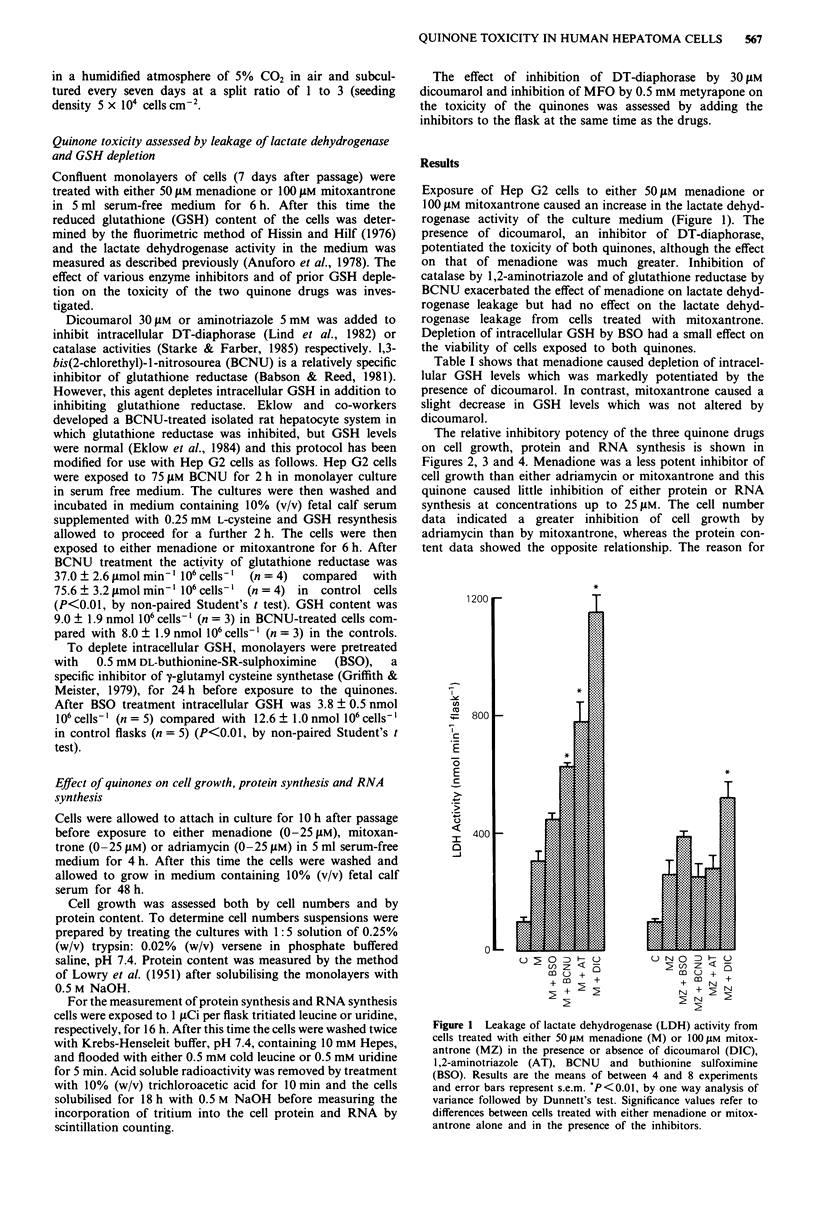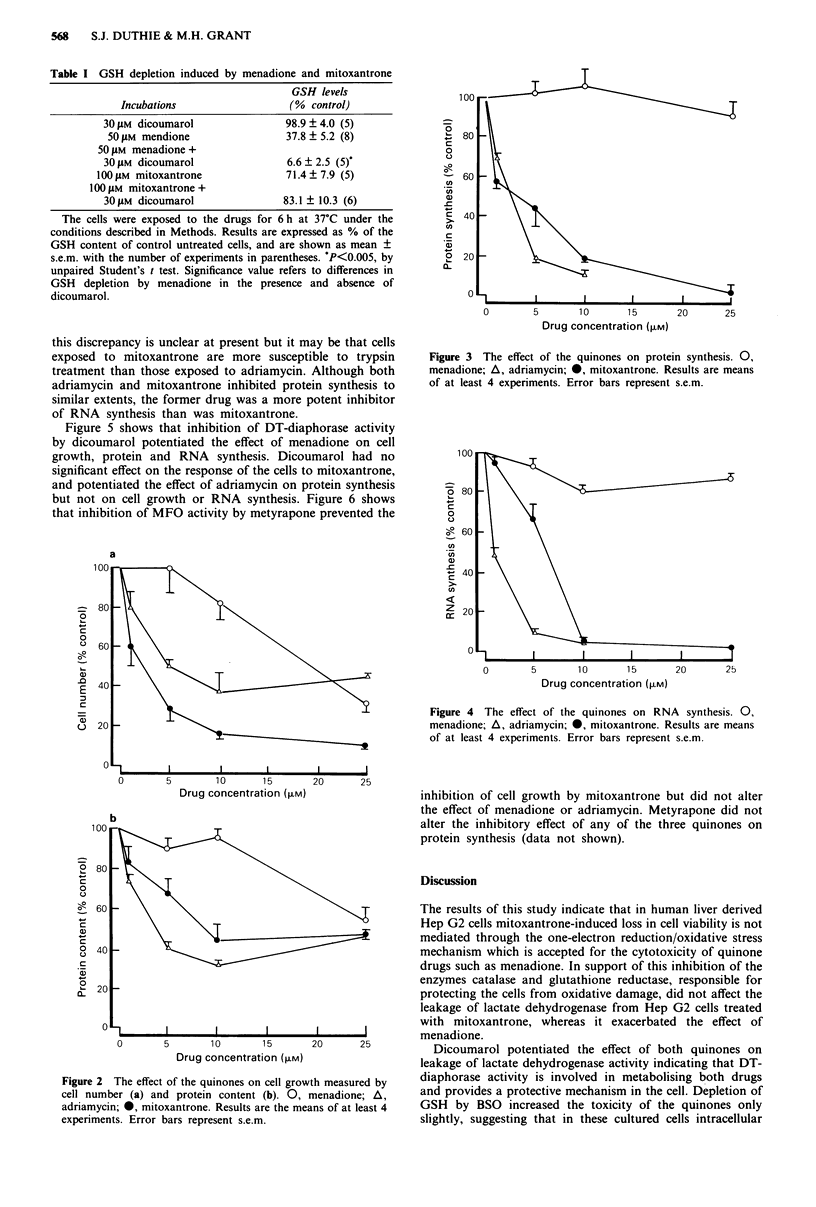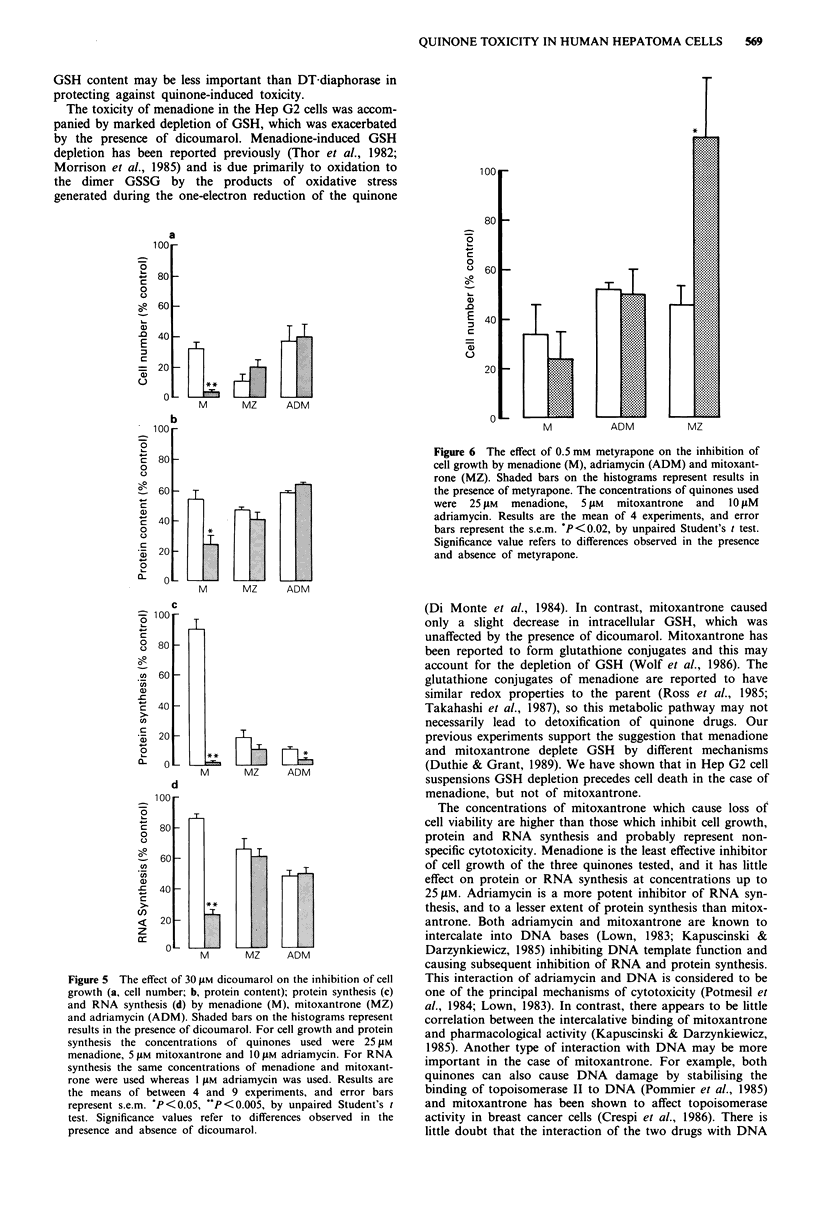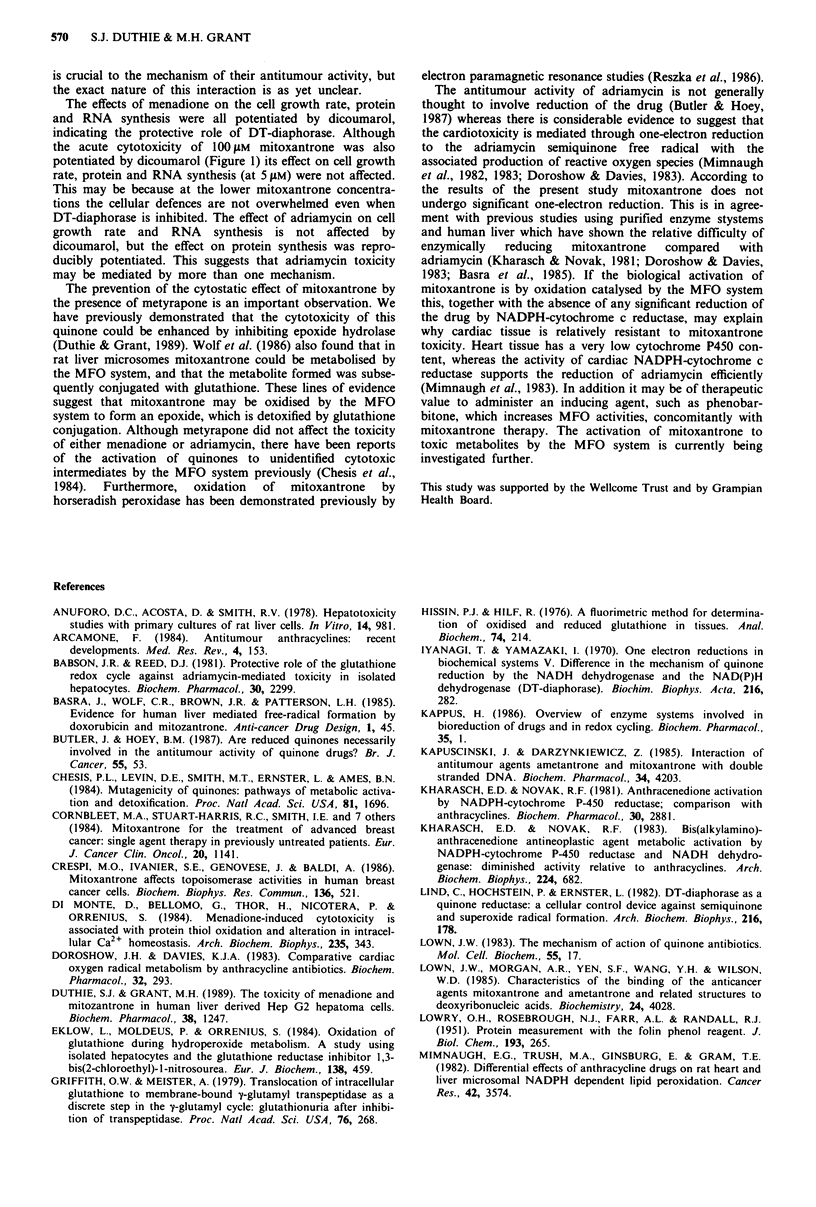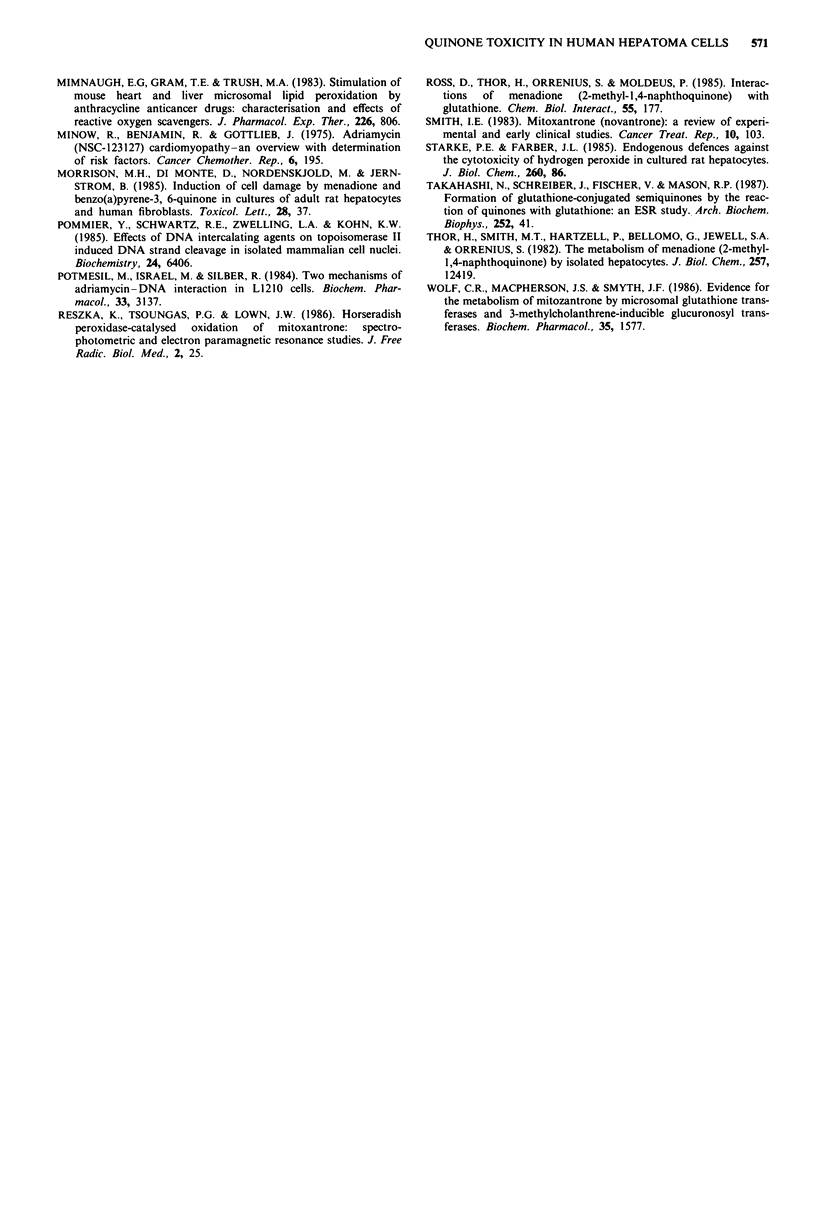# The role of reductive and oxidative metabolism in the toxicity of mitoxantrone, adriamycin and menadione in human liver derived Hep G2 hepatoma cells.

**DOI:** 10.1038/bjc.1989.314

**Published:** 1989-10

**Authors:** S. J. Duthie, M. H. Grant

**Affiliations:** Department of Medicine and Therapeutics, University of Aberdeen, UK.

## Abstract

The cytotoxic properties of quinones, such as menadione, are mediated through one electron reduction to yield semi-quinone radicals which can subsequently enter redox cycles with molecular oxygen leading to the formation of reactive oxygen radicals. In this study the role of reduction and oxidation in the toxicity of mitoxantrone was studied and its toxicity compared with that of adriamycin and menadione. The acute toxicity of mitoxantrone was not mediated through one-electron reduction, since inhibition of the enzymes glutathione reductase and catalase, responsible for protecting the cells against oxidative damage, did not affect its toxicity. Adriamycin was the most potent inhibitor of protein and RNA synthesis of the three quinones. Menadione, at concentrations up to 25 microM, did not inhibit either protein or RNA synthesis unless dicoumarol, an inhibitor of DT-diaphorase, was also present. The two-electron reduction of menadione by DT-diaphorase is therefore a protective mechanism in the cell. This enzyme also protected against the toxicity of high concentrations (100 microM) of mitoxantrone. The inhibitory effect of mitoxantrone, but not of menadione or adriamycin, on cell growth was prevented by inhibiting the activity of cytochrome P450-dependent mixed function oxidase (MFO) system using metyrapone. This suggests that mitoxantrone is oxidised to a toxic intermediate by the MFO system.


Br. J. Cancer (1989), 60, 566-571 © The Macmillan Press Ltd., 1989

### The role of reductive and oxidative metabolism in the toxicity of mitoxantrone, adriamycin and menadione in human liver derived Hep G2 hepatoma cells

S.J. Duthie & M.H. Grant

*Clinical Pharmacology Unit, Department of Medicine and Therapeutics, University of Aberdeen, Polwarth Building, Foresterhill, Aberdeen AB9 2ZD, UK.*

**Summary** The cytotoxic properties of quinones, such as menadione, are mediated through one electron reduction to yield semi-quinone radicals which can subsequently enter redox cycles with molecular oxygen leading to the formation of reactive oxygen radicals. In this study the role of reduction and oxidation in the toxicity of mitoxantrone was studied and its toxicity compared with that of adriamycin and menadione. The acute toxicity of mitoxantrone was not mediated through one-electron reduction, since inhibition of the enzymes glutathione reductase and catalase, responsible for protecting the cells against oxidative damage, did not affect its toxicity. Adriamycin was the most potent inhibitor of protein and RNA synthesis of the three quinones. Menadione, at concentrations up to 25 $\mu$M, did not inhibit either protein or RNA synthesis unless dicoumarol, an inhibitor of DT-diaphorase, was also present. The two-electron reduction of menadione by DT-diaphorase is therefore a protective mechanism in the cell. This enzyme also protected against the toxicity of high concentrations (100 $\mu$M) of mitoxantrone. The inhibitory effect of mitoxantrone, but not of menadione or adriamycin, on cell growth was prevented by inhibiting the activity of cytochrome P450-dependent mixed function oxidase (MFO) system using metyrapone. This suggests that mitoxantrone is oxidised to a toxic intermediate by the MFO system.

The quinoid anthracycline drug adriamycin is one of the most widely prescribed drugs in the treatment of a wide range of human malignancies (Arcamone, 1984). However, the cumulative dose-dependent cardiomyopathy produced by the drug severely restricts its therapeutic usefulness (Minow *et al.*, 1975) and this problem has lead to the development of new structurally related anthraquinone drugs. One of these is mitoxantrone, which is currently used in advanced breast cancer and acute leukaemias (Cornbleet *et al.*, 1984). Although mitoxantrone does not exhibit the same broad spectrum of anti-tumour activity as does adriamycin, the incidence and severity of cardiotoxicity is markedly reduced (Smith, 1983).

The mechanism of action of mitoxantrone is uncertain. There is evidence to suggest that, in common with the anthracyclines, nucleic acids are among the principal cellular targets of the drug (Lown *et al.*, 1985). Since mitoxantrone contains a quinone functional group within its structure it has been considered to undergo activation by metabolic reduction similar to adriamycin (Mimnaugh *et al.*, 1982; Kharasch & Novak, 1983). However, evidence exists which indicates that mitoxantrone is metabolised by cytochrome P-450 dependent mixed function oxidase (MFO) to yield a reactive intermediate which may contribute to its biological activity (Wolf *et al.*, 1986). In this study we have investigated the role of reduction and oxidation in the activation of mitoxantrone to toxic reactive metabolites in human liver derived Hep G2 hepatoma cells using specific enzyme inhibitors. The toxicity of mitoxantrone has been compared with that of the model quinone drug, menadione (2-methyl-1, 4-naphthoquinone), which is known to be mediated by reductive metabolism leading to the formation of semiquinone radicals and reactive oxygen species (Thor *et al.*, 1982).

The metabolism of quinones can proceed by either one-electron reduction to semiquinone radicals or by two-electron reduction to yield the more stable hydroquinones (Iyanagi & Yamazaki, 1970). The latter pathway is considered to be a detoxifying process and is catalysed by NAD(P)H: (quinone acceptor) oxidoreductase (also known as DT-diaphorase) (Lind *et al.*, 1982). One electron reduction is carried out mainly by the action of the enzymes NADPH-cytochrome c reductase and NADH cytochrome $b_5$ reductase (Iyanagi & Yamazaki, 1970). The semiquinones generated by one-electron reduction participate in deleterious redox cycling with molecular oxygen resulting in the formation of various reactive oxygen species, such as superoxide anion, hydrogen peroxide, hydroxyl radical and singlet oxygen. This redox cycling may ultimately lead to oxidative stress and cell toxicity. For instance, Thor *et al.* (1982) observed that exposure of isolated rat hepatocytes to menadione resulted in the production of superoxide, oxidation of glutathione and loss of cell viability. Cellular protection against the effects of reactive oxygen species is provided by the activities of the enzymes glutathione reductase, glutathione peroxidase, catalase and superoxide dismutase (Kappus, 1986).

The aim of this study was to investigate the mechanism(s) responsible for activation of mitoxantrone to cytotoxic metabolites and compare its toxicity with that of menadione and adriamycin. The results provide evidence that mitoxantrone may be activated to a toxic metabolite by cytochrome P450 dependent oxidation and that one-electron reduction is not involved in the mechanism of toxicity. The relevance of these results to improving chemotherapy regimes is discussed.

### Materials and methods

### *Materials*

Reduced glutathione, dicoumarol, metyrapone and 1,2-aminotriazole were obtained from Sigma. Fetal calf serum was from Gibco and Dulbecco's modification of Eagle's medium was from Flow Laboratories. Mitoxantrone was a generous gift from Lederle Laboratories; adriamycin from Farmitalia Carlo Erba and 1,3-*bis*(2-chloroethyl)-1-nitrosourea from Bristol Myers. Hep G2 cells were obtained by Dr W.T. Melvin, Department of Biochemistry, Aberdeen University, from Professor C.N. Hales, Department of Clinical Biochemistry, Addenbrooke's Hospital, Cambridge.

### *Culture of Hep G2 cells*

Hep G2 cells were routinely grown in monolayer or multilayer culture in Dulbecco's modified Eagle's medium supplemented with 10% (v/v) fetal calf serum. They were grown

Correspondence: M.H. Grant, Department of Physiology and Pharmacology, University of Strathclyde, Royal College, 204 George Street, Glasgow G1 1XW, UK.
Received 17 January 1989; and in revised form 19 April 1989.

in a humidified atmosphere of 5% $CO_2$ in air and subcultured every seven days at a split ratio of 1 to 3 (seeding density $5 \times 10^4$ cells $cm^{-2}$.

*Quinone toxicity assessed by leakage of lactate dehydrogenase and GSH depletion*

Confluent monolayers of cells (7 days after passage) were treated with either 50 μM menadione or 100 μM mitoxantrone in 5 ml serum-free medium for 6 h. After this time the reduced glutathione (GSH) content of the cells was determined by the fluorimetric method of Hissin and Hilf (1976) and the lactate dehydrogenase activity in the medium was measured as described previously (Anuforo *et al.*, 1978). The effect of various enzyme inhibitors and of prior GSH depletion on the toxicity of the two quinone drugs was investigated.

Dicoumarol 30 μM or aminotriazole 5 mM was added to inhibit intracellular DT-diaphorase (Lind *et al.*, 1982) or catalase activities (Starke & Farber, 1985) respectively. 1,3-*bis*(2-chlorethyl)-1-nitrosourea (BCNU) is a relatively specific inhibitor of glutathione reductase (Babson & Reed, 1981). However, this agent depletes intracellular GSH in addition to inhibiting glutathione reductase. Eklow and co-workers developed a BCNU-treated isolated rat hepatocyte system in which glutathione reductase was inhibited, but GSH levels were normal (Eklow *et al.*, 1984) and this protocol has been modified for use with Hep G2 cells as follows. Hep G2 cells were exposed to 75 μM BCNU for 2 h in monolayer culture in serum free medium. The cultures were then washed and incubated in medium containing 10% (v/v) fetal calf serum supplemented with 0.25 mM L-cysteine and GSH resynthesis allowed to proceed for a further 2 h. The cells were then exposed to either menadione or mitoxantrone for 6 h. After BCNU treatment the activity of glutathione reductase was $37.0 \pm 2.6$ μmol $min^{-1}$ $10^6$ $cells^{-1}$ ($n = 4$) compared with $75.6 \pm 3.2$ μmol $min^{-1}$ $10^6$ $cells^{-1}$ ($n = 4$) in control cells ($P<0.01$, by non-paired Student's $t$ test). GSH content was $9.0 \pm 1.9$ nmol $10^6$ $cells^{-1}$ ($n = 3$) in BCNU-treated cells compared with $8.0 \pm 1.9$ nmol $10^6$ $cells^{-1}$ ($n = 3$) in the controls.

To deplete intracellular GSH, monolayers were pretreated with 0.5 mM DL-buthionine-SR-sulphoximine (BSO), a specific inhibitor of γ-glutamyl cysteine synthetase (Griffith & Meister, 1979), for 24 h before exposure to the quinones. After BSO treatment intracellular GSH was $3.8 \pm 0.5$ nmol $10^6$ $cells^{-1}$ ($n = 5$) compared with $12.6 \pm 1.0$ nmol $10^6$ $cells^{-1}$ in control flasks ($n = 5$) ($P<0.01$, by non-paired Student's $t$ test).

*Effect of quinones on cell growth, protein synthesis and RNA synthesis*

Cells were allowed to attach in culture for 10 h after passage before exposure to either menadione (0–25 μM), mitoxantrone (0–25 μM) or adriamycin (0–25 μM) in 5 ml serum-free medium for 4 h. After this time the cells were washed and allowed to grow in medium containing 10% (v/v) fetal calf serum for 48 h.

Cell growth was assessed both by cell numbers and by protein content. To determine cell numbers suspensions were prepared by treating the cultures with 1:5 solution of 0.25% (w/v) trypsin: 0.02% (w/v) versene in phosphate buffered saline, pH 7.4. Protein content was measured by the method of Lowry *et al.* (1951) after solubilising the monolayers with 0.5 M NaOH.

For the measurement of protein synthesis and RNA synthesis cells were exposed to 1 μCi per flask tritiated leucine or uridine, respectively, for 16 h. After this time the cells were washed twice with Krebs-Henseleit buffer, pH 7.4, containing 10 mM Hepes, and flooded with either 0.5 mM cold leucine or 0.5 mM uridine for 5 min. Acid soluble radioactivity was removed by treatment with 10% (w/v) trichloroacetic acid for 10 min and the cells solubilised for 18 h with 0.5 M NaOH before measuring the incorporation of tritium into the cell protein and RNA by scintillation counting.

The effect of inhibition of DT-diaphorase by 30 μM dicoumarol and inhibition of MFO by 0.5 mM metyrapone on the toxicity of the quinones was assessed by adding the inhibitors to the flask at the same time as the drugs.

### Results

Exposure of Hep G2 cells to either 50 μM menadione or 100 μM mitoxantrone caused an increase in the lactate dehydrogenase activity of the culture medium (Figure 1). The presence of dicoumarol, an inhibitor of DT-diaphorase, potentiated the toxicity of both quinones, although the effect on that of menadione was much greater. Inhibition of catalase by 1,2-aminotriazole and of glutathione reductase by BCNU exacerbated the effect of menadione on lactate dehydrogenase leakage but had no effect on the lactate dehydrogenase leakage from cells treated with mitoxantrone. Depletion of intracellular GSH by BSO had a small effect on the viability of cells exposed to both quinones.

Table I shows that menadione caused depletion of intracellular GSH levels which was markedly potentiated by the presence of dicoumarol. In contrast, mitoxantrone caused a slight decrease in GSH levels which was not altered by dicoumarol.

The relative inhibitory potency of the three quinone drugs on cell growth, protein and RNA synthesis is shown in Figures 2, 3 and 4. Menadione was a less potent inhibitor of cell growth than either adriamycin or mitoxantrone and this quinone caused little inhibition of either protein or RNA synthesis at concentrations up to 25 μM. The cell number data indicated a greater inhibition of cell growth by adriamycin than by mitoxantrone, whereas the protein content data showed the opposite relationship. The reason for

**Figure 1** Leakage of lactate dehydrogenase (LDH) activity from cells treated with either 50 μM menadione (M) or 100 μM mitoxantrone (MZ) in the presence or absence of dicoumarol (DIC), 1,2-aminotriazole (AT), BCNU and buthionine sulfoximine (BSO). Results are the means of between 4 and 8 experiments and error bars represent s.e.m. *$P<0.01$, by one way analysis of variance followed by Dunnett's test. Significance values refer to differences between cells treated with either menadione or mitoxantrone alone and in the presence of the inhibitors.

**Table I** GSH depletion induced by menadione and mitoxantrone

| *Incubations* | *GSH levels (% control)* |
|---|---|
| 30 μM dicoumarol | 98.9 ± 4.0 (5) |
| 50 μM mendione | 37.8 ± 5.2 (8) |
| 50 μM menadione + 30 μM dicoumarol | 6.6 ± 2.5 (5)* |
| 100 μM mitoxantrone | 71.4 ± 7.9 (5) |
| 100 μM mitoxantrone + 30 μM dicoumarol | 83.1 ± 10.3 (6) |

The cells were exposed to the drugs for 6 h at 37°C under the conditions described in Methods. Results are expressed as % of the GSH content of control untreated cells, and are shown as mean ± s.e.m. with the number of experiments in parentheses. *$P<0.005$, by unpaired Student's *t* test. Significance value refers to differences in GSH depletion by menadione in the presence and absence of dicoumarol.

**Figure 3** The effect of the quinones on protein synthesis. O, menadione; Δ, adriamycin; ●, mitoxantrone. Results are means of at least 4 experiments. Error bars represent s.e.m.

this discrepancy is unclear at present but it may be that cells exposed to mitoxantrone are more susceptible to trypsin treatment than those exposed to adriamycin. Although both adriamycin and mitoxantrone inhibited protein synthesis to similar extents, the former drug was a more potent inhibitor of RNA synthesis than was mitoxantrone.

Figure 5 shows that inhibition of DT-diaphorase activity by dicoumarol potentiated the effect of menadione on cell growth, protein and RNA synthesis. Dicoumarol had no significant effect on the response of the cells to mitoxantrone, and potentiated the effect of adriamycin on protein synthesis but not on cell growth or RNA synthesis. Figure 6 shows that inhibition of MFO activity by metyrapone prevented the inhibition of cell growth by mitoxantrone but did not alter the effect of menadione or adriamycin. Metyrapone did not alter the inhibitory effect of any of the three quinones on protein synthesis (data not shown).

**Figure 4** The effect of the quinones on RNA synthesis. O, menadione; Δ, adriamycin; ●, mitoxantrone. Results are means of at least 4 experiments. Error bars represent s.e.m.

**Figure 2** The effect of the quinones on cell growth measured by cell number (**a**) and protein content (**b**). O, menadione; Δ, adriamycin; ●, mitoxantrone. Results are the means of at least 4 experiments. Error bars represent s.e.m.

### Discussion

The results of this study indicate that in human liver derived Hep G2 cells mitoxantrone-induced loss in cell viability is not mediated through the one-electron reduction/oxidative stress mechanism which is accepted for the cytotoxicity of quinone drugs such as menadione. In support of this inhibition of the enzymes catalase and glutathione reductase, responsible for protecting the cells from oxidative damage, did not affect the leakage of lactate dehydrogenase from Hep G2 cells treated with mitoxantrone, whereas it exacerbated the effect of menadione.

Dicoumarol potentiated the effect of both quinones on leakage of lactate dehydrogenase activity indicating that DT-diaphorase activity is involved in metabolising both drugs and provides a protective mechanism in the cell. Depletion of GSH by BSO increased the toxicity of the quinones only slightly, suggesting that in these cultured cells intracellular

GSH content may be less important than DT-diaphorase in protecting against quinone-induced toxicity.

The toxicity of menadione in the Hep G2 cells was accompanied by marked depletion of GSH, which was exacerbated by the presence of dicoumarol. Menadione-induced GSH depletion has been reported previously (Thor *et al.*, 1982; Morrison *et al.*, 1985) and is due primarily to oxidation to the dimer GSSG by the products of oxidative stress generated during the one-electron reduction of the quinone (Di Monte *et al.*, 1984). In contrast, mitoxantrone caused only a slight decrease in intracellular GSH, which was unaffected by the presence of dicoumarol. Mitoxantrone has been reported to form glutathione conjugates and this may account for the depletion of GSH (Wolf *et al.*, 1986). The glutathione conjugates of menadione are reported to have similar redox properties to the parent (Ross *et al.*, 1985; Takahashi *et al.*, 1987), so this metabolic pathway may not necessarily lead to detoxification of quinone drugs. Our previous experiments support the suggestion that menadione and mitoxantrone deplete GSH by different mechanisms (Duthie & Grant, 1989). We have shown that in Hep G2 cell suspensions GSH depletion precedes cell death in the case of menadione, but not of mitoxantrone.

**Figure 5** The effect of 30 μM dicoumarol on the inhibition of cell growth (**a**, cell number; **b**, protein content); protein synthesis (**c**) and RNA synthesis (**d**) by menadione (M), mitoxantrone (MZ) and adriamycin (ADM). Shaded bars on the histograms represent results in the presence of dicoumarol. For cell growth and protein synthesis the concentrations of quinones used were 25 μM menadione, 5 μM mitoxantrone and 10 μM adriamycin. For RNA synthesis the same concentrations of menadione and mitoxantrone were used whereas 1 μM adriamycin was used. Results are the means of between 4 and 9 experiments, and error bars represent s.e.m. $^*P<0.05$, $^{**}P<0.005$, by unpaired Student's *t* test. Significance values refer to differences observed in the presence and absence of dicoumarol.

**Figure 6** The effect of 0.5 mM metyrapone on the inhibition of cell growth by menadione (M), adriamycin (ADM) and mitoxantrone (MZ). Shaded bars on the histograms represent results in the presence of metyrapone. The concentrations of quinones used were 25 μM menadione, 5 μM mitoxantrone and 10 μM adriamycin. Results are the mean of 4 experiments, and error bars represent the s.e.m. $^*P<0.02$, by unpaired Student's *t* test. Significance value refers to differences observed in the presence and absence of metyrapone.

The concentrations of mitoxantrone which cause loss of cell viability are higher than those which inhibit cell growth, protein and RNA synthesis and probably represent non-specific cytotoxicity. Menadione is the least effective inhibitor of cell growth of the three quinones tested, and it has little effect on protein or RNA synthesis at concentrations up to 25 μM. Adriamycin is a more potent inhibitor of RNA synthesis, and to a lesser extent of protein synthesis than mitoxantrone. Both adriamycin and mitoxantrone are known to intercalate into DNA bases (Lown, 1983; Kapuscinski & Darzynkiewicz, 1985) inhibiting DNA template function and causing subsequent inhibition of RNA and protein synthesis. This interaction of adriamycin and DNA is considered to be one of the principal mechanisms of cytotoxicity (Potmesil *et al.*, 1984; Lown, 1983). In contrast, there appears to be little correlation between the intercalative binding of mitoxantrone and pharmacological activity (Kapuscinski & Darzynkiewicz, 1985). Another type of interaction with DNA may be more important in the case of mitoxantrone. For example, both quinones can also cause DNA damage by stabilising the binding of topoisomerase II to DNA (Pommier *et al.*, 1985) and mitoxantrone has been shown to affect topoisomerase activity in breast cancer cells (Crespi *et al.*, 1986). There is little doubt that the interaction of the two drugs with DNA

is crucial to the mechanism of their antitumour activity, but the exact nature of this interaction is as yet unclear.

The effects of menadione on the cell growth rate, protein and RNA synthesis were all potentiated by dicoumarol, indicating the protective role of DT-diaphorase. Although the acute cytotoxicity of 100 μM mitoxantrone was also potentiated by dicoumarol (Figure 1) its effect on cell growth rate, protein and RNA synthesis (at 5 μM) were not affected. This may be because at the lower mitoxantrone concentrations the cellular defences are not overwhelmed even when DT-diaphorase is inhibited. The effect of adriamycin on cell growth rate and RNA synthesis is not affected by dicoumarol, but the effect on protein synthesis was reproducibly potentiated. This suggests that adriamycin toxicity may be mediated by more than one mechanism.

The prevention of the cytostatic effect of mitoxantrone by the presence of metyrapone is an important observation. We have previously demonstrated that the cytotoxicity of this quinone could be enhanced by inhibiting epoxide hydrolase (Duthie & Grant, 1989). Wolf *et al.* (1986) also found that in rat liver microsomes mitoxantrone could be metabolised by the MFO system, and that the metabolite formed was subsequently conjugated with glutathione. These lines of evidence suggest that mitoxantrone may be oxidised by the MFO system to form an epoxide, which is detoxified by glutathione conjugation. Although metyrapone did not affect the toxicity of either menadione or adriamycin, there have been reports of the activation of quinones to unidentified cytotoxic intermediates by the MFO system previously (Chesis *et al.*, 1984). Furthermore, oxidation of mitoxantrone by horseradish peroxidase has been demonstrated previously by electron paramagnetic resonance studies (Reszka *et al.*, 1986).

The antitumour activity of adriamycin is not generally thought to involve reduction of the drug (Butler & Hoey, 1987) whereas there is considerable evidence to suggest that the cardiotoxicity is mediated through one-electron reduction to the adriamycin semiquinone free radical with the associated production of reactive oxygen species (Mimnaugh *et al.*, 1982, 1983; Doroshow & Davies, 1983). According to the results of the present study mitoxantrone does not undergo significant one-electron reduction. This is in agreement with previous studies using purified enzyme stystems and human liver which have shown the relative difficulty of enzymically reducing mitoxantrone compared with adriamycin (Kharasch & Novak, 1981; Doroshow & Davies, 1983; Basra *et al.*, 1985). If the biological activation of mitoxantrone is by oxidation catalysed by the MFO system this, together with the absence of any significant reduction of the drug by NADPH-cytochrome c reductase, may explain why cardiac tissue is relatively resistant to mitoxantrone toxicity. Heart tissue has a very low cytochrome P450 content, whereas the activity of cardiac NADPH-cytochrome c reductase supports the reduction of adriamycin efficiently (Mimnaugh *et al.*, 1983). In addition it may be of therapeutic value to administer an inducing agent, such as phenobarbitone, which increases MFO activities, concomitantly with mitoxantrone therapy. The activation of mitoxantrone to toxic metabolites by the MFO system is currently being investigated further.

This study was supported by the Wellcome Trust and by Grampian Health Board.

### References

ANUFORO, D.C., ACOSTA, D. & SMITH, R.V. (1978). Hepatotoxicity studies with primary cultures of rat liver cells. *In Vitro*, **14**, 981.

ARCAMONE, F. (1984). Antitumour anthracyclines: recent developments. *Med. Res. Rev.*, **4**, 153.

BABSON, J.R. & REED, D.J. (1981). Protective role of the glutathione redox cycle against adriamycin-mediated toxicity in isolated hepatocytes. *Biochem. Pharmacol.*, **30**, 2299.

BASRA, J., WOLF, C.R., BROWN, J.R. & PATTERSON, L.H. (1985). Evidence for human liver mediated free-radical formation by doxorubicin and mitozantrone. *Anti-cancer Drug Design*, **1**, 45.

BUTLER, J. & HOEY, B.M. (1987). Are reduced quinones necessarily involved in the antitumour activity of quinone drugs? *Br. J. Cancer*, **55**, 53.

CHESIS, P.L., LEVIN, D.E., SMITH, M.T., ERNSTER, L. & AMES, B.N. (1984). Mutagenicity of quinones: pathways of metabolic activation and detoxification. *Proc. Natl Acad. Sci. USA*, **81**, 1696.

CORNBLEET, M.A., STUART-HARRIS, R.C., SMITH, I.E. and 7 others (1984). Mitoxantrone for the treatment of advanced breast cancer: single agent therapy in previously untreated patients. *Eur. J. Cancer Clin. Oncol.*, **20**, 1141.

CRESPI, M.O., IVANIER, S.E., GENOVESE, J. & BALDI, A. (1986). Mitoxantrone affects topoisomerase activities in human breast cancer cells. *Biochem. Biophys. Res. Commun.*, **136**, 521.

DI MONTE, D., BELLOMO, G., THOR, H., NICOTERA, P. & ORRENIUS, S. (1984). Menadione-induced cytotoxicity is associated with protein thiol oxidation and alteration in intracellular $Ca^{2+}$ homeostasis. *Arch. Biochem. Biophys.*, **235**, 343.

DOROSHOW, J.H. & DAVIES, K.J.A. (1983). Comparative cardiac oxygen radical metabolism by anthracycline antibiotics. *Biochem. Pharmacol.*, **32**, 293.

DUTHIE, S.J. & GRANT, M.H. (1989). The toxicity of menadione and mitozantrone in human liver derived Hep G2 hepatoma cells. *Biochem. Pharmacol.*, **38**, 1247.

EKLOW, L., MOLDEUS, P. & ORRENIUS, S. (1984). Oxidation of glutathione during hydroperoxide metabolism. A study using isolated hepatocytes and the glutathione reductase inhibitor 1,3-bis(2-chloroethyl)-1-nitrosourea. *Eur. J. Biochem.*, **138**, 459.

GRIFFITH, O.W. & MEISTER, A. (1979). Translocation of intracellular glutathione to membrane-bound γ-glutamyl transpeptidase as a discrete step in the γ-glutamyl cycle: glutathionuria after inhibition of transpeptidase. *Proc. Natl Acad. Sci. USA*, **76**, 268.

HISSIN, P.J. & HILF, R. (1976). A fluorimetric method for determination of oxidised and reduced glutathione in tissues. *Anal. Biochem.*, **74**, 214.

IYANAGI, T. & YAMAZAKI, I. (1970). One electron reductions in biochemical systems V. Difference in the mechanism of quinone reduction by the NADH dehydrogenase and the NAD(P)H dehydrogenase (DT-diaphorase). *Biochim. Biophys. Acta*, **216**, 282.

KAPPUS, H. (1986). Overview of enzyme systems involved in bioreduction of drugs and in redox cycling. *Biochem. Pharmacol.*, **35**, 1.

KAPUSCINSKI, J. & DARZYNKIEWICZ, Z. (1985). Interaction of antitumour agents ametantrone and mitoxantrone with double stranded DNA. *Biochem. Pharmacol.*, **34**, 4203.

KHARASCH, E.D. & NOVAK, R.F. (1981). Anthracenedione activation by NADPH-cytochrome P-450 reductase; comparison with anthracyclines. *Biochem. Pharmacol.*, **30**, 2881.

KHARASCH, E.D. & NOVAK, R.F. (1983). Bis(alkylamino)-anthracenedione antineoplastic agent metabolic activation by NADPH-cytochrome P-450 reductase and NADH dehydrogenase: diminished activity relative to anthracyclines. *Arch. Biochem. Biophys.*, **224**, 682.

LIND, C., HOCHSTEIN, P. & ERNSTER, L. (1982). DT-diaphorase as a quinone reductase: a cellular control device against semiquinone and superoxide radical formation. *Arch. Biochem. Biophys.*, **216**, **178**.

LOWN, J.W. (1983). The mechanism of action of quinone antibiotics. *Mol. Cell. Biochem.*, **55**, 17.

LOWN, J.W., MORGAN, A.R., YEN, S.F., WANG, Y.H. & WILSON, W.D. (1985). Characteristics of the binding of the anticancer agents mitoxantrone and ametantrone and related structures to deoxyribonucleic acids. *Biochemistry*, **24**, 4028.

LOWRY, O.H., ROSEBROUGH, N.J., FARR, A.L. & RANDALL, R.J. (1951). Protein measurement with the folin phenol reagent. *J. Biol. Chem.*, **193**, 265.

MIMNAUGH, E.G., TRUSH, M.A., GINSBURG, E. & GRAM, T.E. (1982). Differential effects of anthracycline drugs on rat heart and liver microsomal NADPH dependent lipid peroxidation. *Cancer Res.*, **42**, 3574.

MIMNAUGH, E.G, GRAM, T.E. & TRUSH, M.A. (1983). Stimulation of mouse heart and liver microsomal lipid peroxidation by anthracycline anticancer drugs: characterisation and effects of reactive oxygen scavengers. *J. Pharmacol. Exp. Ther.*, **226**, 806.

MINOW, R., BENJAMIN, R. & GOTTLIEB, J. (1975). Adriamycin (NSC-123127) cardiomyopathy–an overview with determination of risk factors. *Cancer Chemother. Rep.*, **6**, 195.

MORRISON, M.H., DI MONTE, D., NORDENSKJOLD, M. & JERNSTROM, B. (1985). Induction of cell damage by menadione and benzo(a)pyrene-3, 6-quinone in cultures of adult rat hepatocytes and human fibroblasts. *Toxicol. Lett.*, **28**, 37.

POMMIER, Y., SCHWARTZ, R.E., ZWELLING, L.A. & KOHN, K.W. (1985). Effects of DNA intercalating agents on topoisomerase II induced DNA strand cleavage in isolated mammalian cell nuclei. *Biochemistry*, **24**, 6406.

POTMESIL, M., ISRAEL, M. & SILBER, R. (1984). Two mechanisms of adriamycin–DNA interaction in L1210 cells. *Biochem. Pharmacol.*, **33**, 3137.

RESZKA, K., TSOUNGAS, P.G. & LOWN, J.W. (1986). Horseradish peroxidase-catalysed oxidation of mitoxantrone: spectrophotometric and electron paramagnetic resonance studies. *J. Free Radic. Biol. Med.*, **2**, 25.

ROSS, D., THOR, H., ORRENIUS, S. & MOLDEUS, P. (1985). Interactions of menadione (2-methyl-1,4-naphthoquinone) with glutathione. *Chem. Biol. Interact.*, **55**, 177.

SMITH, I.E. (1983). Mitoxantrone (novantrone): a review of experimental and early clinical studies. *Cancer Treat. Rep.*, **10**, 103.

STARKE, P.E. & FARBER, J.L. (1985). Endogenous defences against the cytotoxicity of hydrogen peroxide in cultured rat hepatocytes. *J. Biol. Chem.*, **260**, **86**.

TAKAHASHI, N., SCHREIBER, J., FISCHER, V. & MASON, R.P. (1987). Formation of glutathione-conjugated semiquinones by the reaction of quinones with glutathione: an ESR study. *Arch. Biochem. Biophys.*, **252**, 41.

THOR, H., SMITH, M.T., HARTZELL, P., BELLOMO, G., JEWELL, S.A. & ORRENIUS, S. (1982). The metabolism of menadione (2-methyl-1,4-naphthoquinone) by isolated hepatocytes. *J. Biol. Chem.*, **257**, 12419.

WOLF, C.R., MACPHERSON, J.S. & SMYTH, J.F. (1986). Evidence for the metabolism of mitozantrone by microsomal glutathione transferases and 3-methylcholanthrene-inducible glucuronosyl transferases. *Biochem. Pharmacol.*, **35**, 1577.